# Normothermic Machine and Regional Perfusion in US DCD Liver Transplantation: A National Comparative Analysis Supporting Adoption as Standard of Care

**DOI:** 10.1097/SLA.0000000000007120

**Published:** 2026-06-23

**Authors:** Sergio A. Acuna, Hiren Dayala, Maggie E. Jones-Carr, Paul MacLennan, M. Chandler McLeod, Devin E. Eckhoff, Robert M. Cannon

**Affiliations:** Department of Surgery, Division of Transplantation, Heersink School of Medicine, University of Alabama at Birmingham, Birmingham, AL

**Keywords:** donation after circulatory death, liver transplantation, normothermic regional perfusion, normothermic machine perfusion, UNOS/OPTN registry analysis

## Abstract

**Objective:**

To compare the effectiveness in US practice of normothermic regional perfusion (NRP) and normothermic machine perfusion (NMP) for donation after circulatory death (DCD) liver transplantation (LT).

**Background:**

DCD livers historically conferred inferior outcomes to donation after brain death donors, primarily due to ischemic biliary injury. NRP and NMP have emerged as promising strategies to mitigate this risk.

**Methods:**

Using UNOS data (2022-2024), we stratified DCD donors by procurement [super-rapid recovery (SRR) or NRP] and preservation [static cold storage (SCS) or NMP] method. NMP cases were further stratified into on-site versus back-to-base initiation of perfusion. On-site NMP was directly captured in the UNOS dataset. NRP and back-to-base NMP were identified with surrogate markers. We then compared graft (GS) and overall (OS) survival in propensity-matched cohorts.

**Results:**

We identified 4632 DCD LTs. The most common procurement-preservation strategy was SRR-NMP (2637; 56.9%), followed by SRR-SCS (937; 20.2%), NRP-SCS (560; 12.1%), and NRP-NMP (498; 10.8%). SRR-NMP demonstrated superior GS (HR: 0.54, 95% CI: 0.32–0.92) versus SRR-SCS. NRP-SCS similarly reduced the risk of graft loss (HR: 0.42, 95% CI: 0.29–0.61) versus SRR-SCS. NRP-SCS and SRR-NMP had similar GS. Similarly, adding NMP to livers procured with NRP did not lead to improved outcomes compared with NRP followed by SCS.

**Conclusions:**

This study provides the most comprehensive US comparison of modern procurement and preservation strategies in DCD LT. Both NRP and NMP were associated with improved GS versus the historical standard (SRR-SCS). No significant survival differences were observed between NRP and NMP, confirming that these strategies serve complementary roles in DCD LT. These findings support the adoption of both NRP and NMP as the new standard of care in DCD LT.

Maximizing the use of deceased donor livers is essential to address the persistent gap between organ availability and the demand for liver transplantation (LT). Donation after circulatory death (DCD) grafts provide an opportunity to expand the donor pool; however, these organs are uniquely vulnerable to ischemic injury during standard rapid recovery (SRR), static cold storage (SCS), and reperfusion.^1,2^ Early enthusiasm for DCD LT was tempered by reports of high biliary complication rates and inferior graft survival (GS) compared with donation after brain death.^3,4^

The introduction of normothermic perfusion technologies has transformed the field. In situ normothermic regional perfusion (NRP) and ex situ normothermic machine perfusion (NMP) both mitigate ischemia-reperfusion injury and improve transplant outcomes, leading some to suggest that these techniques should represent the standard of care for DCD LT.^5^ Randomized clinical trials have demonstrated that NMP preserves livers in a near-physiologic condition, reduces early allograft dysfunction, and facilitates viability assessment; however, these studies applied restrictive donor and recipient criteria and included relatively few DCD grafts, limiting generalizability.^6–8^ Meanwhile, excellent outcomes reported with NRP in European series have prompted rapid adoption in the United States, with early US experiences confirming comparable benefits.^9–11^ Although often considered competing strategies, NRP and NMP are better conceptualized as complementary: NRP is a procurement method that addresses ischemic injury by early restoration of perfusion after the initial warm ischemia interval, whereas NMP is a preservation method that maintains physiologic conditions for the graft, allowing prolonged storage and ongoing functional assessment of organ viability.

Several important questions remain unresolved. The comparative efficacy of NRP and NMP compared with SRR-SCS and to each other has yet to be examined in a large cohort outside a clinical trial. The incremental value of applying NMP following NRP has not been established, with evidence largely limited to small studies and case reports.^12^ In practice, combined use typically occurs when organs recovered with NRP are allocated to centers that routinely employ NMP. Similarly, it is unclear whether NMP performed on-site at the donor hospital is equivalent to “back-to-base” initiation after transport to the transplant center using SCS. A small, randomized trial suggested that back-to-base NMP maintains the benefits of perfusion, but available data are limited.^13^

Limited preservation data in national databases remains a challenge to studying the application of NRP and NMP. Investigators have attempted to infer use of these techniques from United Network for Organ Sharing/Organ Procurement and Transplant Network (UNOS/OPTN) data.^14,15^ For example, bimodal distributions of death-to-aortic cross-clamp times have been used as a surrogate to identify NRP utilization,^14^ while bimodal distributions of cold ischemia times among livers not coded as receiving NMP suggest misclassification of grafts that actually underwent back-to-base NMP.^15^ Building on these methods, the present study provides the first comprehensive evaluation of NRP and NMP in a real-world setting using national UNOS/OPTN data.

## METHODS

### Data source and study population

We conducted a retrospective, propensity score–matched cohort study of all patients who underwent LT with DCD grafts in the United States between January 1, 2022, and December 31, 2024, using data from the United Network for Organ Sharing/Organ Procurement and Transplant Network (UNOS/OPTN) database. We excluded recipients below 18 years of age, partial grafts, any prior solid organ transplantation, and multiorgan transplants. Patients were followed for overall and GS for 1 year after transplant.

### Exposure classification

NRP and back-to-base NMP are not captured in the UNOS/OPTN database. Death-to-cross-clamp time was derived using the donor time of death and cross-clamp time. The distribution of the derived death-to-cross-clamp time was examined visually, confirming a bimodal pattern as previously documented (Supplemental Figure 1, Supplemental Digital Content 1, http://links.lww.com/SLA/F863).^14^ Donors with times <30 minutes were classified as SRR, those with DCC ≥40 minutes were classified as undergoing NRP, and those with times between 30 and 40 minutes (n=6) were categorized as being in a “washout” period and excluded. Because UNOS/OPTN only captures NMP applied at the donor hospital, back-to-base NMP cases are misclassified as not receiving machine perfusion.^15^ As with the NRP definition, the distribution of preservation time was examined visually, confirming a bimodal pattern as previously documented (Supplemental Figure 2, Supplemental Digital Content 1, http://links.lww.com/SLA/F863). Patients with preservation time ≥10 hours were then reclassified as receiving back-to-base NRP. It should be noted that a limitation of the UNOS/OPTN data is that the variable for “cold ischemia time” actually reflects total preservation time, without differentiation between true cold ischemia and time spent on an NMP device. For example, a liver that is preserved on NMP for 24 hours would have a “cold ischemia time” of 24 hours recorded in the STAR file. Observations with missing or implausible donor death-to-cross-clamp times (negative values or >12 h) were excluded. Cases with missing exposure classification were excluded.

### Propensity score matching

We used propensity score matching to control for differences in donor, recipient, and transplant center-related factors as we have previously described.^16^ Propensity scores were estimated using logistic regression, and a complete list of all variables utilized to estimate propensity scores is provided in the Supplemental Methods, Supplemental Digital Content 1, http://links.lww.com/SLA/F863. Matching was then performed with the Mayo Clinic GMATCH macro, using 1:1 greedy nearest-neighbor matching without replacement and a caliper width of 0.2 SDs of the logit of the propensity score.^17^ Balance of covariates after matching was assessed using standardized mean differences (SMDs), with an SMD <0.1 in absolute value considered evidence of adequate balance between groups (Supplemental Figure 3, Supplemental Digital Content 1, http://links.lww.com/SLA/F863). Propensity score distributions before and after matching were visually inspected (Supplemental Figure 4, Supplemental Digital Content 1, http://links.lww.com/SLA/F863).

### Group comparisons

We first compared NRP and NMP with the historical standard of care (SRR and SCS). Next, we compared NRP with NMP and evaluated whether outcomes differed between NRP followed by SCS versus NRP followed by NMP. Finally, we assessed whether on-site NMP and back-to-base NMP were associated with different outcomes by comparing them with each other and with the standard of care (SCS).

### Statistical analysis

Continuous variables were summarized as means with SDs and compared using the Student *t* test. Categorical variables were summarized as counts and percentages and compared using the χ^2^ test; when expected cell counts were <5, Fisher exact test was applied, with Monte Carlo simulation used for variables with >2 categories and small expected cell counts. Overall survival (OS) and GS in propensity-matched cohorts were estimated using Kaplan-Meier methods, and survival functions were compared using log-rank tests. Hazard ratios (HRs) with 95% CIs were calculated using Cox proportional hazards models, with robust variance estimates applied to account for the matched nature of the data. The regression models were adjusted for variables whose SMD were >0.1. All analyses were conducted using SAS 9.4 (SAS Institute) and R version 4.5.1 (R Foundation for Statistical Computing). *P* value <0.05 was considered statistically significant.

## RESULTS

**FIGURE 1:**
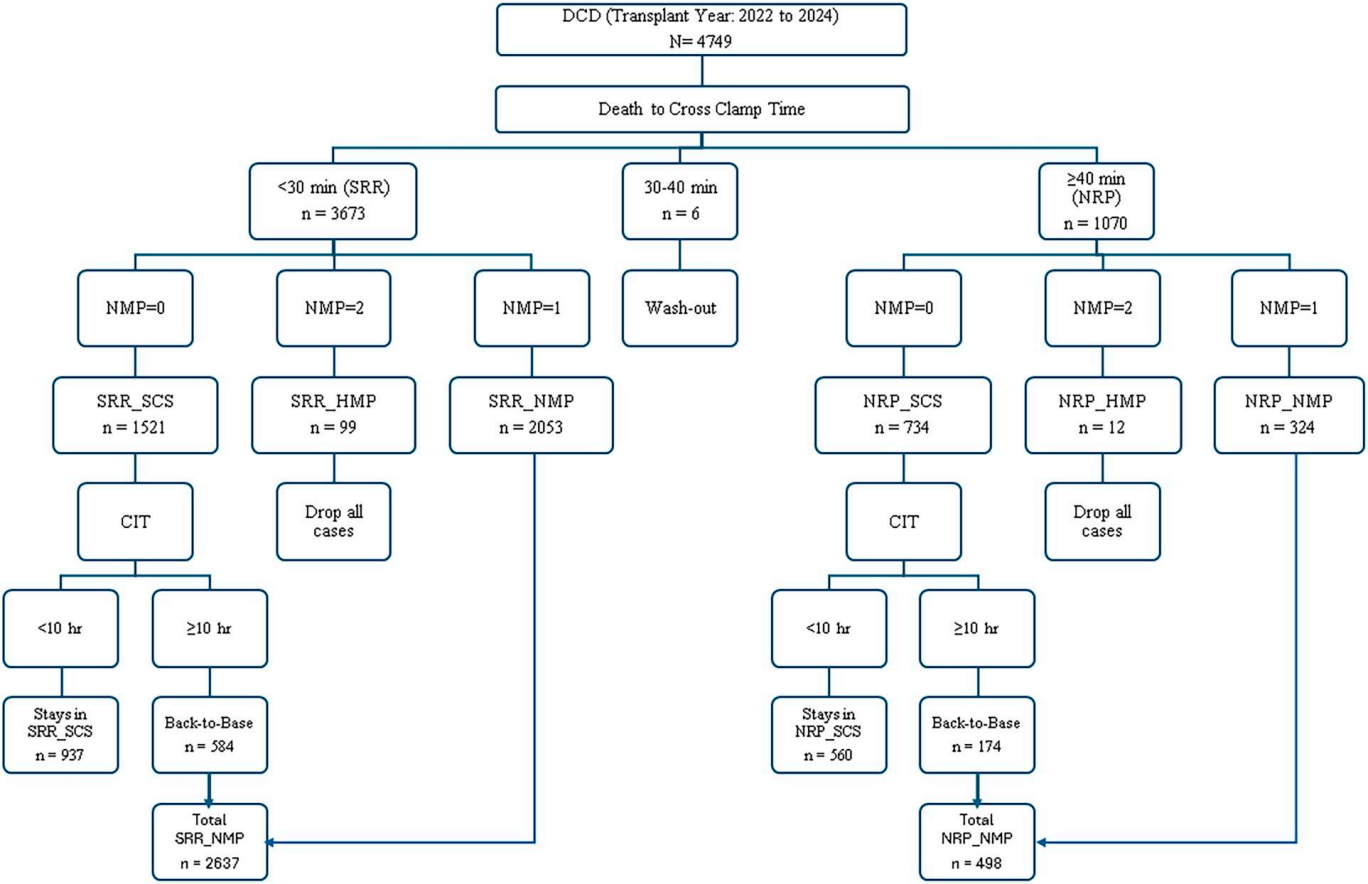
Flowchart of cohort allocation to perfusion strategies.

There were 5767 patients who underwent DCD liver transplant in the United States during the study period. After applying the following exclusion criteria: recipient age below 18 years, partial grafts, previous liver or multiorgan transplants, primary diagnosis of graft failure and missing brain death or time, the cohort included 4749 patients. In addition, exclusions included 99 SRR with HMP cases, 12 NRP with HMP cases, and 6 cases with death-to-cross-clamp time between 30 and 40 minutes. Out of 4632 patients undergoing DCD LT comprising the final cohort, most grafts were procured using SRR and NMP (2637; 56.9%), followed by SRR with SCS (937; 20.2%), NRP with SCS (560; 12.1%) and NRP with NMP (498; 10.8%) as shown in Figure 1. Compared with grafts procured using SRR with SCS, donor undergoing SRR and NMP were older, had more diabetes, hypertension, macrosteatosis, higher BMIs and liver DSRI scores, and the grafts traveled longer distances (Table 1). Similar donor patterns were observed when comparing SRR with SCS and NRP with SCS donors (Table 1).

**TABLE 1:** Baseline Recipient and Donor Characteristics by SRR-SCS, SRR-NMP, and NRP-SCS in DCD Liver Transplantation, United States, 2022-2024

Variables	SRR_SCS (n=937)	SRR_NMP (n=2637)	*P* ^*^	NRP_SCS (n=560)	*P* ^†^
Recipient Factors
Age	57.13 (9.98)	56.75 (10.85)	0.33	56.28 (10.39)	0.12
Height (cm)	172.68 (10.47)	171.78 (10.33)	**0.02**	172.59 (10.57)	0.87
Weight (kg)	86.95 (19.74)	86.66 (20.2)	0.71	88.06 (20.42)	0.30
BMI (kg/m^2^)	29.1 (5.88)	29.27 (5.93)	0.44	29.46 (5.79)	0.25
Calculated MELD Score	18.73 (6.74)	19.12 (7.51)	0.14	19.33 (6.82)	0.10
Cold ischemic time (h)	5.61 (1.62)	16.82 (5.68)	**<0.01**	5.13 (1.57)	**<0.01**
Gender (%)			0.38		0.38
Male	614 (65.5)	1664 (63.1)		371 (66.3)	
Female	323 (34.5)	973 (36.9)		189 (33.8)	
Ethnicity (%)			**<0.01**		**<0.01**
Caucasian	722 (77.1)	1885 (71.5)		420 (75.0)	
African American	39 (4.2)	100 (3.8)		21 (3.8)	
Hispanic	138 (14.7)	467 (17.7)		84 (15.0)	
Asian	26 (2.8)	86 (3.3)		10 (1.8)	
Native American/other	12 (1.3)	99 (3.8)		25 (4.5)	
ABO			0.58		0.58
A	367 (39.2)	1041 (39.5)		232 (41.4)	
AB	26 (2.8)	73 (2.8)		7 (1.3)	
B	78 (8.3)	232 (8.8)		48 (8.6)	
O	466 (49.7)	1291 (49.0)		273 (48.8)	
Diabetes			**<0.01**		**<0.01**
Yes	265 (28.3)	890 (33.8)		155 (27.7)	
No	672 (71.7)	1747 (66.2)		405 (72.3)	
Portal vein thrombosis			0.09		0.10
Yes	123 (13.1)	397 (15.1)		84 (15.0)	
No	814 (86.9)	2232 (84.6)		474 (84.6)	
Unknown	0 (0.0)	8 (0.3)		2 (0.4)	
Ascites at transplant			0.58		0.58
None	214 (22.8)	609 (23.1)		109 (19.5)	
Mild	431 (46.0)	1243 (47.1)		278 (49.6)	
Moderate	292 (31.2)	785 (29.8)		173 (30.9)	
Encephalopathy at transplant			0.90		0.90
Grade 1–2	848 (90.5)	2379 (90.2)		507 (90.5)	
Grade 3–4	89 (9.5)	258 (9.8)		53 (9.5)	
Previous abdominal surgery			0.06		0.06
Yes	494 (52.7)	1425 (54.0)		275 (49.1)	
No	432 (46.1)	1166 (44.2)		277 (49.5)	
Unknown	11 (1.2)	46 (1.7)		8 (1.4)	
On dialysis at transplant			0.08		0.14
Yes	15 (1.6)	72 (2.7)		7 (1.3)	
No	916 (97.8)	2553 (96.8)		553 (98.8)	
Unknown	6 (0.6)	12 (0.5)		0 (0.0)	
On ventilator at transplant			0.54		0.33
Yes	2 (0.2)	12 (0.5)		3 (0.5)	
* *No	935 (99.8)	2625 (99.5)		557 (99.5)	
History of spontaneous bacterial peritonitis			0.84		0.47
Yes	88 (9.4)	260 (9.9)		55 (9.8)	
No	849 (90.6)	2375 (90.1)		504 (90.0)	
Unknown	0 (0.0)	2 (0.1)		1 (0.2)	
Medical condition at transplant			**0.04**		**0.04**
Home	804 (85.8)	2205 (83.6)		487 (87.0)	
Hospitalized	115 (12.3)	341 (12.9)		59 (10.5)	
ICU	18 (1.9)	91 (3.5)		13 (2.3)	
MELD exception status			**<0.01**		**<0.01**
No exception	677 (72.3)	2569 (97.4)		522 (93.2)	
HCC exception	37 (3.9)	17 (0.6)		7 (1.3)	
Non-HCC exception	223 (23.8)	51 (1.9)		32(5.7)	
Hepatitis C NAT			0.21		0.89
Positive	34 (3.6)	69 (2.6)		21 (3.8)	
Negative	903 (96.4)	2567 (97.3)		539 (96.3)	
Unknown	0 (0.0)	1 (0.0)		0 (0.0)	
Working for Income			**<0.01**		**<0.01**
Yes	271 (28.9)	879 (33.3)		199 (35.5)	
No	649 (69.3)	1739 (65.9)		360 (64.3)	
Unknown	17 (1.8)	19 (0.7)		1 (0.2)	
TIPS shunt			**<0.01**		**<0.01**
Yes	124 (13.2)	313 (11.9)		55 (9.8)	
No	797 (85.1)	2200 (83.4)		491 (87.7)	
Unknown	16 (1.7)	124 (4.7)		14 (2.5)	
Diagnosis at transplant			**0.01**		0.47
Acute liver failure	2 (0.2)	14 (0.5)		1 (0.2)	
Alcohol cirrhosis	317 (33.8)	792 (30.0)		188 (33.6)	
Alcohol hepatitis	21 (2.2)	88 (3.3)		15 (2.7)	
Cholestatic disease	47 (5.0)	191 (7.2)		36 (6.4)	
HCV	51 (5.4)	98 (3.7)		17 (3.0)	
MASH	201 (21.5)	560 (21.2)		121 (21.6)	
Malignancy	188 (20.1)	548 (20.8)		111 (19.8)	
Other	110 (11.7)	346 (13.1)		71 (12.7)	
Donor factors					
Age	39.96 (13.44)	47.15 (13.95)	**<0.01**	46.81 (14.27)	**<0.01**
Height	171.53 (10.52)	170.66 (11.1)	**0.03**	172.51 (10.33)	0.08
Weight	80.3 (19.21)	84.76 (21.98)	**<0.01**	85.7 (21.67)	**<0.01**
BMI	27.28 (6.17)	29.1 (7.05)	**<0.01**	28.87 (7.27)	**<0.01**
AST	74.44 (102.54)	77.44 (141.38)	0.49	79.01 (124.07)	0.46
ALT	76.14 (128.84)	73.51 (164.47)	0.62	68.66 (104.11)	0.22
Total bilirubin	0.68 (0.55)	0.64 (0.54)	0.10	0.78 (2.41)	0.32
Liver DSRI	34.96 (36.53)	51.4 (71.69)	**<0.01**	53.33 (75.43)	**<0.01**
Distance	129.17 (178.14)	213.75 (289.02)	**<0.01**	103.02 (153.53)	**<0.01**
Gender			0.37		0.37
Male	610 (65.1)	1668 (63.3)		370 (66.1)	
Female	327 (34.9)	969 (36.7)		190 (33.9)	
Ethnicity			**<0.01**		**<0.01**
Caucasian	680 (72.6)	1943 (73.7)		428 (76.4)	
African American	148 (15.8)	304 (11.5)		65 (11.6)	
Hispanic	75 (8.0)	277 (10.5)		50 (8.9)	
Asian	25 (2.7)	59 (2.2)		7 (1.3)	
Native American/other	9 (1.0)	54 (2.0)		10 (1.8)	
ABO			0.52		0.91
A	379 (40.4)	1028 (39.0)		228 (40.7)	
AB	9 (1.0)	44 (1.7)		4 (0.7)	
B	82 (8.8)	226 (8.6)		44 (7.9)	
O	467 (49.8)	1339 (50.8)		284 (50.7)	
Diabetes			**<0.01**		**<0.01**
Yes	98 (10.5)	467 (17.7)		98 (17.5)	
No	828 (88.4)	2148 (81.5)		459 (82.0)	
Unknown	11 (1.2)	22 (0.8)		3 (0.5)	
Cause of death			0.32		0.94
Anoxia	506 (54.0)	1382 (52.4)		295 (52.7)	
CVA	199 (21.2)	622 (23.6)		111 (19.8)	
Trauma	190 (20.3)	491 (18.6)		125 (22.3)	
Other	42 (4.5)	142 (5.4)		29 (5.2)	
IV drug use			0.28		0.28
Yes	108 (11.5)	256 (9.7)		65 (11.6)	
No	815 (87.0)	2331 (88.4)		483 (86.3)	
Unknown	14 (1.5)	50 (1.9)		12 (2.1)	
HIV NAT			0.16		1.00
Positive	1 (0.1)	0 (0.0)		0 (0.0)	
Negative	936 (99.9)	2634 (99.9)		560 (100.0)	
Unknown	0 (0.0)	3 (0.1)		0 (0.0)	
Hepatitis C NAT			0.21		0.89
Positive	34 (3.6)	69 (2.6)		21 (3.8)	
Negative	903 (96.4)	2567 (97.3)		539 (96.3)	
Unknown	0 (0.0)	1 (0.0)		0 (0.0)	
Heavy alcohol use			**0.02**		**0.02**
Yes	174 (18.6)	566 (21.5)		145 (25.9)	
No	734 (78.3)	1975 (74.9)		396 (70.7)	
Unknown	29 (3.1)	96 (3.6)		19 (3.4)	
History of hypertension			**<0.01**		**<0.01**
Positive	293 (31.3)	1179 (44.7)		247 (44.1)	
Negative	633 (67.6)	1433 (54.3)		306 (54.6)	
Unknown	11 (1.2)	25 (0.9)		7 (1.3)	
Macrosteatosis			**<0.01**		**<0.01**
No biopsy	717 (76.5)	1336 (50.7)		293 (52.3)	
1 (<30)	209 (22.3)	1232 (46.7)		255 (45.5)	
2(≥30)	11 (1.2)	69 (2.6)		12 (2.1)	

Continuous variables are presented as mean±SD and compared using Student *t* test.

Categorical variables as n (%) and compared using χ^2^ or Fisher exact test, where appropriate.

^*^ *P* value compares SRR_NMP versus SRR_SCS. Values <0.05 indicate statistical significance.

^†^ *P* value compares NRP_SCS versus SRR_SCS. Values < 0.05 indicate statistical significance.

NRP_SCS indicates normothermic regional perfusion with static cold storage; SRR_NMP, super-rapid recovery with normothermic machine perfusion; SRR_SCS, super-rapid recovery with static cold storage.

### SRR-NMP versus SRR-SCS

**FIGURE 2:**
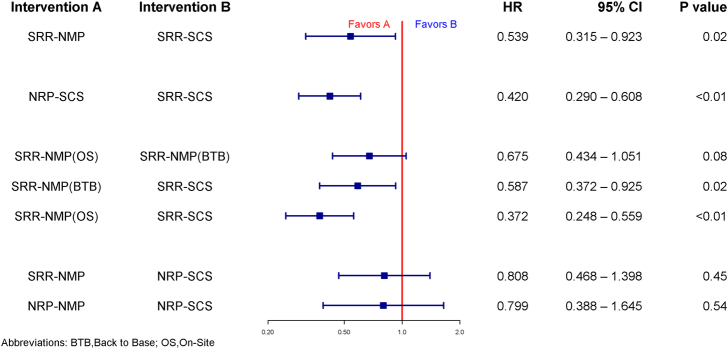
Graft survival comparison among different perfusion strategies.

**FIGURE 3:**
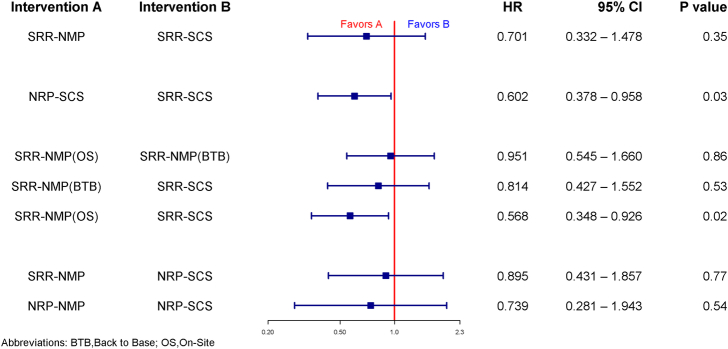
Overall survival comparison among different perfusion strategies.

After propensity score matching (n=586 per group), baseline characteristics (Table 1) were well balanced (Supplemental Figure 3A, Supplemental Digital Content 1, http://links.lww.com/SLA/F863). NMP-preserved grafts after SRR were associated with superior GS (HR: 0.54, 95% CI: 0.32–0.92, Fig. 2) and OS (HR: 0.70, 95% CI: 0.33–0.48, Fig. 3) compared with SCS.

### SRR-NMP on-site versus SRR-NMP back-to-base

A total of 580 grafts undergoing NMP on-site were propensity score matched to 580 grafts undergoing NMP back-to-base preservation (Supplemental Table 1, Supplemental Figure 3B, Supplemental Digital Content 1, http://links.lww.com/SLA/F863). Similarly, 527 grafts undergoing NMP on-site were propensity score matched to 527 grafts undergoing SCS, and 350 grafts undergoing NMP back-to-base were matched to 350 grafts undergoing SCS (Supplemental Table 1, Supplemental Figures 3C, 3D, Supplemental Digital Content 1, http://links.lww.com/SLA/F863). No differences in graft and OS were observed between NMP on-site versus back-to-base after SRR (Figs. 2, 3). However, when compared individually to SRR-SCS, NMP on-site was associated with improved graft (HR: 0.37, 95% CI: 0.29–0.56, Fig. 2) and OS (HR: 0.59, 95% CI: 0.35–0.93, Fig. 3) while NMP back-to-base was associated with improved GS, but not OS (GS HR: 0.59, 95% CI: 0.37–0.93, OS HR: 0.81, 95% CI: 0.43–1.55).

### NRP-SCS versus SRR-SCS

When comparing NRP-SCS to SRR-SCS (307 matched pairs, Supplemental Figure 3E, Supplemental Digital Content 1, http://links.lww.com/SLA/F863), NRP was associated with improved graft (HR: 0.42, 95% CI: 0.29–0.61, Fig. 2) and OS (HR: 0.6, 95% CI: 0.38–0.96, Fig. 3).

### NRP-SCS versus SRR-NMP

When comparing NRP-SCS to SRR-NMP (538 matched pairs, Supplemental Figure 3F, Supplemental Digital Content 1, http://links.lww.com/SLA/F863), there were no differences in graft or OS (GS HR: 0.81, 95% CI: 0.47–1.4, OS HR: 0.9, 95% CI: 0.43–1.86, Figs. 2, 3).

### NRP-SCS versus NRP-NMP

Finally, NRP-SCS was compared with NRP-NMP in 368 matched pairs (Supplemental Table 2, Supplemental Figure 3G, Supplemental Digital Content 1, http://links.lww.com/SLA/F863). The addition of NMP to NRP was not associated with improved graft (HR: 0.80, 95% CI: 0.39–1.65, Fig. 2) or OS (HR: 0.74, 95% CI: 0.28–1.94, Fig. 3).

## DISCUSSION

In this large US registry study of DCD LT recipients, we demonstrate that both NRP and NMP are associated with significantly improved graft and patient survival compared with conventional SRR-SCS. Importantly, by applying novel classification algorithms to identify NRP and back-to-base NMP within the UNOS/OPTN dataset, this study overcomes long-standing limitations of registry data and enables a large-scale comparison of these emerging technologies across real-world practice.^15^ The findings confirm the growing body of evidence supporting normothermic perfusion as a transformative advance in DCD liver utilization and suggest that NRP and NMP are associated with comparable outcomes through distinct but complementary mechanisms.

Prior randomized clinical trials and prospective registries have demonstrated that NMP preserves livers in a near-physiologic condition, reduces early allograft dysfunction, and facilitates viability assessment, leading to improved graft outcomes compared with SCS.^6–8,18^ However, these studies primarily enrolled standard-risk donors, limiting their generalizability.^18^ The present analysis extends these findings to real-world US practice, showing that NMP is associated with survival advantages even in an unselected national cohort. Similarly, prior reports from European and single-center US experiences have consistently shown that NRP mitigates the deleterious effects of warm ischemia and reduces ischemic cholangiopathy and graft failure.^9,10^ This study confirms those benefits in a national cohort and, importantly, demonstrates that both NMP and NRP are associated with improved graft and patient survival compared with standard rapid recovery and SCS in DCD LT.

Although often conceptualized as alternative approaches, NRP and NMP exert their benefits at distinct phases of the donation-transplant continuum and serve complementary roles. NRP restores physiological perfusion rapidly after cessation of circulation, limiting the cumulative duration of warm ischemia and enabling in situ functional recovery of the graft before cold preservation.^19^ NMP, in contrast, preserves the liver in a near-physiologic state ex situ, allowing for prolonged storage and ongoing functional assessment before implantation.^7,8^ The comparable outcomes observed in this study suggest that each strategy may optimize different components of the ischemia-reperfusion sequence rather than compete directly. The absence of a clear advantage for combined NRP + NMP use supports the hypothesis that most of the ischemic injury occurs before or during the early reperfusion phase, and that once physiologic conditions are restored, whether in situ or ex situ, further benefit is incremental.

This study also provides some evidence on the long-standing question of whether back-to-base NMP, initiated after graft transport to the recipient center using SCS, achieves equivalent outcomes to on-site initiation at the donor hospital. Direct comparison of SRR-NMP on-site and SRR-NMP back to base demonstrated no statistically significant differences in either graft or patient survival. When on-site NMP and back-to-base NMP were individually compared to rapid recovery with SCS, both techniques were associated with improved GS; however, only on-site NMP was associated with improved patient survival. The sole prior available data comparing on-site to back-to-base NMP is a nonrandomized pilot study of 46 liver grafts, of which only 10 were DCDs, that found no difference in 30-day graft and OS.^13^ This pilot study is limited in both the duration of follow-up and the power to detect clinically meaningful differences. Consistent with the premise that delays between cross-clamp/initial cooling and NMP reperfusion worsen graft outcomes, a multicenter cohort of 384 DCD LT found that a prolonged interval from cross-clamp to on-site NMP start (>2 h 45 min) was associated with a higher incidence of early allograft dysfunction.^20^ This observation underscores the physiological rationale that NMP restores the liver to a near-physiologic oxygenated state, facilitating metabolic recovery. Therefore, prolonged cold ischemia before the onset of normothermic reperfusion, as occurs in back-to-base NMP, may attenuate these protective effects and contribute to inferior graft recovery. The strength of these conclusions for on-site versus back-to-base NMP is weakened by limitations of our study design, in which prolonged preservation time is used as a surrogate for back-to-base NMP use. Although this approach is supported by the current literature, the risk of ascertainment bias remains substantial. As an example, a team encountering unexpected difficulties in the recipient hepatectomy after accepting a DCD liver for SRR-SCS may inadvertently end up with a preservation time of over 10 hours. This prolonged true cold ischemia would be expected to substantially increase the risk for graft loss. Because our method would incorrectly identify liver as undergoing back-to-base NMP, the results of our study would thus be biased toward the null hypothesis of benefit for back-to-base NMP. The question of superiority for on-site versus back-to-base NMP will ultimately have to await a direct comparison when the preservation modalities are explicitly identified. This limitation underscores the need for OPTN data collection to be updated to reflect modern procurement and preservation practices.

A major contribution of this study is the introduction of data-driven classification algorithms that enable the identification of perfusion strategies in national transplant data where explicit coding is absent. The use of bimodal distributions of death-to-cross-clamp and cold ischemia times provides a reproducible, transparent approach for differentiating NRP and back-to-base NMP, respectively. These methods build on and refine previous approaches,^14,15^ substantially expanding the ability to evaluate real-world outcomes at scale. Such methodology can be adapted for future analyses as perfusion utilization grows, and coding becomes more standardized within OPTN datasets. In addition, the application of propensity score matching and direct, side-by-side comparison of all major preservation techniques strengthens the validity and interpretability of the observed associations.

This study has several limitations. First, despite robust propensity score matching and sensitivity analyses, residual confounding from unmeasured donor variables such as agonal time, initial warm ischemia time, NRP duration, or time to reperfusion on NMP cannot be excluded. Second, the classification of NRP and NMP was inferential, based on time distributions rather than direct reporting, introducing the possibility of misclassification bias. However, the observed bimodal patterns and consistency with published benchmarks support the validity of the approach. Third, it is possible that the no difference in graft and OS between back-to-base NMP and SCS is secondary to the small sample size for this subgroup. Finally, follow-up was limited to 1 year due to the recency of widespread NRP and NMP adoption in the United States, precluding assessment of long-term graft durability and biliary outcomes.

Beyond clinical outcomes, perfusion strategies may also hold important economic implications. Early biliary complications, particularly strictures, substantially increase post-transplant costs and resource utilization, as demonstrated in prior US cost analyses.^21^ Reductions in ischemic cholangiopathy with both NRP and NMP therefore have the potential to yield meaningful downstream savings alongside improved clinical results.

Collectively, these findings have important implications for clinical practice and policy. The comparable outcomes between NRP and NMP suggest that both technologies can be deployed effectively to expand the DCD donor pool, with selection guided by institutional resources, logistics, and expertise rather than anticipated survival differences. Centers without access to NRP infrastructure may achieve equivalent results through NMP, and vice versa. From a policy perspective, these data support the argument in favor of NRP and NMP as the new standard of care in DCD LT and underscore the need for standardized perfusion reporting within UNOS/OPTN and for prospective studies evaluating long-term outcomes and cost-effectiveness across technologies. Broader adoption of perfusion strategies represents a tangible path toward improving the safe utilization of DCD livers and narrowing the persistent gap between organ availability and demand.

## Supplementary Material

## DISCUSSANT

**Dr. James Guarrera (Philadelphia, PA)** Dr Cannon, you, and your colleagues present an important and timely national analysis of normothermic perfusion strategies in over 5000 DCD liver transplants using UNOS data from 2022 to 2024.

To my knowledge, these 5000 cases are the most comprehensive US comparison of procurement and preservation approaches in the modern perfusion era.

The methodology is very strong with a thoughtful strategy to address the limitations of registry data by employing plausible, data-driven surrogate markers, using bimodal pronouncement to cross-clamp times to identify NRP and cold ischemia distributions to infer back-to-base machine perfusion. These assumptions are transparent and reproducible.

The study is further strengthened by robust propensity score matching across recipient, donor, and center-level variables, resulting in excellent covariate balance across all key comparisons.

This allows meaningful “real-world” assessment of strategies that are often discussed but rarely evaluated side by side at this scale.

From a clinical standpoint, the findings are highly significant and interesting. Both NRP and NMP are associated with markedly improved GS compared with the historical SRR and cold storage controls.

Importantly, the similar outcomes observed between NRP with cold storage and stand-alone NMP suggest these techniques are complementary rather than competing, enabling centers to select strategies based on infrastructure and logistics rather than some perceived superiority.

The observation that only on-site NMP and not back-to-base NMP retains a clear survival advantage over SCS is particularly relevant and may have implications for program design and resource allocation.

That said, the back-to-base NMP strategy provides additional benefits of allowing lengthening of preservation time to facilitate logistics and allow time for viability testing.

Taken together, this work strongly supports your conclusion that normothermic perfusion strategies should be considered the contemporary standard of care for DCD LT; however, several questions arise.

Given that only on-site NMP clearly outperformed SCS, not back-to-base, how should centers without on-site perfusion capability interpret these findings when applying DCD recovery strategies?

Is it possible that specific differences in NMP devices/techniques/protocols, rather than the site of perfusion initiation, may have some role in outcomes?

Considering that you did not observe a survival benefit to adding NMP in NRP cases, in an era of cost containment and increasing scrutiny of resource utilization, how should these data inform policy decisions, reimbursement models, and adoption of sequential perfusion strategies?

Do the authors suggest that their findings shift the balance toward “stand-alone NRP”?

Do you anticipate a wider role for hypothermic machine perfusion as a complementary technique in NRP cases, which has improved outcomes in large series from Europe and other countries and in recent clinical trials in the United States?

**Dr. Robert Cannon (Birmingham, AL)** I’ll address the first question about on-site versus back-to-base NMP.

Honestly, I think we really should n’t draw strong conclusions for our analysis from that standpoint. The registry data does not capture NMP when it is applied in a back-to-base model. So we had to use a surrogate based on preservation time.

I think it’s very likely that the lack of a statistically significant difference for back-to-base NMP versus cold storage is an artifact of this limitation, that is, small numbers and ascertainment bias. We aren’t certain from the data that livers did get put on a pump after being brought back to base.

Second, is a stand-alone NRP adequate? I think it certainly is if you have short cold ischemia time.

Typically, if you’re doing NRP and then going straight in, at least in this cohort, the average cold ischemia time was still under 6 hours.

So while that is certainly beneficial and provides everything you need for patient benefit, adding some sort of perfusion on the back end of NRP can give you the logistical benefits. Say, for example, allow your recipient to wait at home while awaiting for the donor to expire.

NMP is very costly to use in that manner, and the coming introduction of hypothermic machine perfusion, which has very strong evidence from Europe and recent US trials, I think, will be very interesting.

A lot of centers, including ours, plan to start adopting hypothermic perfusion following NRP.

**Dr. Prabhakar Baliga (Charleston, SC)** Have you done any subanalysis based on the changes in allocation of how long livers travel today? Because I think the distance matters in terms of the cold times?

If I have a local donor, whichever way you preserve it does not matter, but if you have a liver coming from outside, it may make a big difference.

In your analysis, have you looked at perhaps livers that cross regions or cross states?

**Dr. Robert Cannon (Birmingham, AL)** We didn’t do subanalysis on outcomes by distance, but we did see differences in travel by method. So both the NRP going straight into transplantation, as well as the standard method, had an average distance of roughly 100 nautical miles, while those undergoing machine perfusion traveled on average 200 nautical miles.

We’ve shown in some previous work that having that additional preservation period has sort of normalized the geographic distribution of liver acceptance.

**Dr. Seth Karp (Nashville, TN)** Our president, Dr Drebin, advised us to know about the finances. Tell me a little bit about what you think the difference in cost is, and how does that scale across the whole United States?

**Dr. Robert Cannon (Birmingham, AL)** So the most expensive technology, easily, is going to be NMP, particularly applied in the on-site fashion.

NRP can be developed more cheaply depending upon if you’re going to develop it with your own center. That’s why we think it’s also going to be very important to consider these technologies as the standard of care rather than experimental.

Payers really ought to be reimbursing for this, and some already are. Medicare will cover these theoretically under the cost report. Anthem Blue Cross allows this in its policy determinations, but it’s a mixed bag.

I think it’s important to consider the cost of this in terms of the alternative to transplantation, however. If you’re transplanting people faster without multiple admissions for cirrhosis-related complications, you ultimately save money for the system in the long run by using these more expensive technologies.

**Dr. William Chapman (St., MO)** I would echo some of the previous comments that the cost differential is substantial.

For programs to maintain a margin, can you discuss some of the details of programs that have had big jumps in the number of transplants, but their margin has disappeared?

Now, maybe that will come back in the future, but I think we’re not there yet. I think you may want to consider that to say NRP at $10,000, or NMP, at least with one of the companies, over $100,000 per case is monumental.

Did you look at the results for kidney recovery in the DCDs with NRP? NRP results in markedly improved kidney function, which you don’t see in rapid recovery NMP donors.

To me, NRP should become our standard, and whether or not you put the liver on NMP for logistic reasons is a separate question.

**Dr. Robert Cannon (Birmingham, AL)** We agree with you wholeheartedly, and that’s why our center is adopting an NRP protocol right now, which will include the use of NMP or HMP on the back end for livers. We didn’t look, in this study, at kidney outcomes. One of my colleagues is looking at NRP kidneys, and she’s going to have some promising results.

**Dr. Tomoaki Kato (New York, NY)** The use of DCD has rapidly increased. Part of the reason is the viability testing that we can do on the pump.

Do you have data that the cases are put on the pump and decline, and how many are included in this analysis?

How many cases are put on the pump but still decline, not used? Do you have data for that? Because of the rapid recovery, you don’t really have viability testing. Once you recover, you put it in.

Second, because of the viability testing, we are becoming more aggressive in the use of DCD. For example, the functional warm ischemic time in the donor is getting longer. We’re accepting more of the cases that we never have accepted in the past.

Do you have data to compare the cases that are put on NMP/NRP versus the previous era standards and techniques in a previous era? Is there any data to suggest that tendency more aggressively, accepting DCD out of the previous criteria?

**Dr. Robert Cannon (Birmingham, AL)** So we don’t have data on livers that were put on a pump and then declined. We were only able to look at patients who were transplanted.

Although from some of the other studies, you see once the liver gets on a pump, there’s a lot of selection bias that goes into that when you’re going to pull that trigger to go on a pump. But very few livers fail on the pump.

In terms of data on warm ischemia time, we don’t have that very well captured in our current regulatory data.

OPTN has a new data collection initiative looking at DCD vital signs that’ll be very helpful.

Yes, a lot of times, almost everyone we know has shifted their warm ischemia criteria to a systolic of 50 or less, as opposed to a historical, more conservative standard of systolic of 80 or even SATs of 80.

Many centers don’t even look at oxygen saturation anymore, but the European data are very promising on expanded acceptance practices for prolonged warm ischemia, particularly from Italy.

**Dr. Peter Abt (Philadelphia, PA)** Are you concerned about misclassification of NMP cases?

There’s some published data suggesting that many of these cases, perhaps up to 20%, are misclassified, particularly when they’re brought back to base to be put on the pump, because there’s not a good mechanism to catch whether these livers are actually pumped or not.

**Dr. Robert Cannon (Birmingham, AL)** I do think there’s almost certainly some misclassification for back-to-base NMP in this study, which is why I wouldn’t draw strong conclusions from those specific comparisons in our study. We used a preservation time cutoff of >10 hours to assume back-to-base.

Not many people would willingly use a DCD with over 10 hours of cold ischemia time on ice with rapid recovery, although it does happen sometimes due to unforeseen circumstances. That would certainly bias the study against the back-to-base. So I would not draw strong conclusions from that particular comparison.

## References

[1] Skaro AI Jay CL Baker TB . The impact of ischemic cholangiopathy in liver transplantation using donors after cardiac death: the untold story. Surgery. 2009;146:543–553. 19789011 10.1016/j.surg.2009.06.052 PMC2790600

[2] Paterno F Guarrera JV Wima K . Clinical implications of donor warm and cold ischemia time in donor after circulatory death liver transplantation. Liver Transpl. 2019;25:1342–1352. 30912253 10.1002/lt.25453

[3] Foley DP Fernandez LA Leverson G . Donation after cardiac death: the University of Wisconsin experience with liver transplantation. Ann Surg. 2005;242:724–731. 16244547 10.1097/01.sla.0000186178.07110.92 PMC1409855

[4] Jay C Ladner D Wang E . A comprehensive risk assessment of mortality following donation after cardiac death liver transplant–an analysis of the national registry. J Hepatol. 2011;55:808–813. 21338639 10.1016/j.jhep.2011.01.040 PMC3177011

[5] Croome KP . Should advanced perfusion be the standard of care for donation after circulatory death liver transplant? Am J Transplant. 2024;24:1127–1131. 38514015 10.1016/j.ajt.2024.03.021

[6] Parente A Tirotta F Pini A . Machine perfusion techniques for liver transplantation—ā meta-analysis of the first seven randomized-controlled trials. J Hepatol. 2023;79:1201–1213. 37302578 10.1016/j.jhep.2023.05.027

[7] Nasralla D Coussios CC Mergental H . A randomized trial of normothermic preservation in liver transplantation. Nature. 2018;557:50–56. 29670285 10.1038/s41586-018-0047-9

[8] Markmann JF Abouljoud MS Ghobrial RM . Impact of portable normothermic blood-based machine perfusion on outcomes of liver transplant: the OCS liver PROTECT randomized clinical trial. JAMA Surgery. 2022;157:189–198. 34985503 10.1001/jamasurg.2021.6781 PMC8733869

[9] Hessheimer AJ de la Rosa G Gastaca M . Abdominal normothermic regional perfusion in controlled donation after circulatory determination of death liver transplantation: outcomes and risk factors for graft loss. Am J Transplant. 2022;22:1169–1181. 34856070 10.1111/ajt.16899

[10] Watson CJ Hunt F Messer S . In situ normothermic perfusion of livers in controlled circulatory death donation may prevent ischemic cholangiopathy and improve graft survival. Am J Transplant. 2019;19:1745–1758. 30589499 10.1111/ajt.15241

[11] Brubaker AL Sellers MT Abt PL . US liver transplant outcomes after normothermic regional perfusion vs standard super rapid recovery. JAMA Surgery. 2024;159:677–685. 38568597 10.1001/jamasurg.2024.0520 PMC10993160

[12] Ghinolfi D Melandro F Torri F . The role of sequential normothermic regional perfusion and end-ischemic normothermic machine perfusion in liver transplantation from very extended uncontrolled donation after cardiocirculatory death. Artif Organs. 2023;47:432–440. 36461895 10.1111/aor.14468

[13] Bral M Dajani K Leon Izquierdo D . A back-to-base experience of human normothermic ex situ liver perfusion: does the chill kill? Liver Transpl. 2019;25:848–858. 30938039 10.1002/lt.25464

[14] Bakhtiyar SS Maksimuk TE Gutowski J . Association of procurement technique with organ yield and cost following donation after circulatory death. Am J Transplant. 2024;24:1803–1815. 38521350 10.1016/j.ajt.2024.03.027

[15] Muñoz N Minus E Abt P . The underreporting of liver machine perfusion in US national data. American Journal of Transplantation. 2025;25:633–634. 39617348 10.1016/j.ajt.2024.11.027

[16] Cannon RM Nassel AF Walker JT . Lost potential and missed opportunities for DCD liver transplantation in the United States. Am J Transplant. 2022;224:990–998. 10.1016/j.amjsurg.2022.05.001 PMC9940905 35589438

[17] GMATCH SAS Macro [computer program] . Version Accessed 2025. Mayo Clinic; 2004.

[18] Ravikumar R Jassem W Mergental H . Liver transplantation after ex vivo normothermic machine preservation: a phase 1 (first-in-man) clinical trial. Am J Transplant. 2016;16:1779–1787. 26752191 10.1111/ajt.13708

[19] Net M Valero R Almenara R . The effect of normothermic recirculation is mediated by ischemic preconditioning in NHBD liver transplantation. Am J Transplant. 2005;5:2385–2392. 16162186 10.1111/j.1600-6143.2005.01052.x

[20] Lee C Mathur AK Mao S . Prolonged time from cross-clamp until normothermic machine perfusion start is associated with an increased risk of early allograft dysfunction following DCD liver transplant. Liver Transpl. 2025;31:616–622. 39652008 10.1097/LVT.0000000000000548

[21] Bhutiani N Jones JM Wei D . A cost analysis of early biliary strictures following orthotopic liver transplantation in the United States. Clin Transplant. 2018;32:e13396. 30160322 10.1111/ctr.13396

