# Supplementary material for: Normothermic Machine and Regional Perfusion in US DCD Liver Transplantation: A National Comparative Analysis Supporting Adoption as Standard of Care
Source: Ann Surg. 2026 Jun 23;284(3):481–91. doi: 10.1097/SLA.0000000000007120 (PMC13460313; doi:10.1097/SLA.0000000000007120)
Supplement: Supplementary file 1 [file sla-284-481-s001.docx]

**Normothermic Machine and Regional Perfusion in U.S. DCD Liver Transplantation:**

**A National Comparative Analysis Supporting Adoption as Standard of Care**

Sergio A. Acuna; Hiren Dayala; Maggie E. Jones-Carr; Paul MacLennan; M. Chandler McLeod; Devin E. Eckhoff; Robert M. Cannon

**Online-Only Supplemental Data Content**

1. **Supplemental Methods**
2. **Supplemental Tables**
3. **Supplemental Figures**

**Supplemental Methods:**

**Covariates for Propensity Matching**

We included the following covariates in the matching and multivariable analyses:

**Recipient characteristics:** Age, body mass index (BMI, kg/m²), calculated MELD score, Preservation time (hours), gender, ethnicity, ABO blood group, diabetes status, portal vein thrombosis, ascites at transplant, encephalopathy at transplant, previous abdominal surgery, dialysis dependence, ventilatory support, history of spontaneous bacterial peritonitis, medical condition at transplant (home, hospitalized, ICU), MELD exception status, Hepatitis C NAT status, working for income, presence of TIPS shunt, and primary diagnosis at transplant (acute liver failure, alcohol cirrhosis, alcoholic hepatitis, cholestatic disease, HCV, MASH, malignancy, other).

**Donor characteristics:** Age, BMI, AST, ALT, total bilirubin, liver donor risk index (DSRI), donor-recipient distance, gender, ethnicity, ABO blood group, diabetes status, cause of death, history of IV drug use, HIV NAT status, Hepatitis C NAT status, heavy alcohol use, history of hypertension, and degree of macrosteatosis (no biopsy, <30%, ≥30%).

**Transplant center characteristics:** Annual liver transplant volume (quartiles) and transplant year.

**Derived Covariates:**

We derived additional variables from the UNOS/OPTN database to support our analysis. Donor risk was quantified using the Liver DSRI as described by Rana et al.^1^ Primary indications for LT were reclassified into clinically meaningful diagnostic groups, including acute liver failure, hepatitis C virus (HCV)–related disease, alcohol-associated hepatitis, alcohol-associated cirrhosis, metabolic dysfunction–associated steatohepatitis (MASH), cholestatic diseases, malignancy (such as hepatocellular carcinoma or cholangiocarcinoma), and other or unknown causes. Donor macrovascular steatosis was categorized as <30% versus ≥30%, with missing biopsy data coded as a separate “no biopsy” category. For each transplant center, DCD liver volume across the study period was calculated, ranked, and stratified into quartiles (Q1–Q4) yielding a center-level volume quartile variable.

**References**

1.	Rana A, Sigireddi RR, Halazun KJ, et al. Predicting liver allograft discard: the discard risk index. *Transplantation* 2018; 102(9):1520–1529.

**Supplemental Figures:**

**Supplemental Figure 1. Distribution of derived death-to-cross-clamp time.**

The distribution of death-to-cross-clamp time was visually examined and confirmed a bimodal pattern.

**Supplemental Figure 2. Distribution of Preservation Time.**

The distribution of preservation time was visually examined and confirmed a bimodal pattern.

**Supplemental Figure 3. Assessment of matching quality using standardized mean differences (SMD).**
Matching quality was evaluated for all covariates before and after propensity score matching across 7 group comparisons. Panels (A)–(G) represent: An SMD <0.1 was considered evidence of adequate balance between groups.

(A) SRR_SCS vs SRR_NMP

(B) NMP (Back-to-Base vs On-site)

(C) SRR (Back-to-Base vs Stays in SCS)

(D) SRR_Stays_SCS vs SRR_NMP_On-site

(E) SRR_SCS vs NRP_SCS

(F) NRP_SCS vs SRR_NMP

(G) NRP_NMP vs NRP_SCS

**Supplemental Figure 4.** **Propensity score distributions before and after matching.**

Propensity score distributions were visually inspected for all seven group comparisons before and after matching. Panels (A)–(G) correspond to the same groups as in **Supplemental** Figure 2, demonstrating improved overlap and balance after matching.

(A) NRP_SCS vs SRR_NMP

 (B) NRP_NMP vs NRP_SCS

(C) SRR_SCS vs SRR_NMP

(D) SRR_SCS vs NRP_SCS

(E) SRR-NMP (Back-to-Base vs On-site)

(F) SRR (Back-to-Base vs Stays in SCS)

(G) SRR_Stays_SCS vs SRR_NMP_On-site

**Supplemental Figure 5. Graft survival after matching across all comparison groups.**

Kaplan–Meier curves demonstrating graft survival for all seven matched comparison groups. Panels (A)–(G) correspond to the same groups as in **Supplemental** Figure 2.

(A) NRP_SCS vs SRR_NMP

(B) NRP_NMP vs NRP_SCS

(C) SRR_SCS vs SRR_NMP

(D) SRR_SCS vs NRP_SCS

(E) NMP (Back-to-Base vs On-site)

(F) SRR (Back-to-Base vs Stays in SCS)

(G) SRR_Stays_SCS vs SRR_NMP_On-site

**Supplemental Figure 6. Patient survival after matching across all comparison groups***.*

Kaplan–Meier curves demonstrating overall patient survival for all seven matched comparison groups. Panels (A)–(G) correspond to the same groups as in **Supplemental** Figure 2.

(A) NRP_SCS vs SRR_NMP

(B) NRP_NMP vs NRP_SCS

(C) SRR_SCS vs SRR_NMP

(D) SRR_SCS vs NRP_SCS

(E) NMP (Back-to-Base vs On-site)

(F) SRR (Back-to-Base vs Stays in SCS)

(G) SRR_Stays_SCS vs SRR_NMP_On-site

**Supplemental Table 1. Baseline recipient and donor characteristics comparing SRR-SCS, SRR-NMP (overall), and SRR-NMP subgroups (On-Site vs Back-**

| **to-Base) in DCD liver transplantation, United States, 2022–2024** | | | | | | | | |
| --- | --- | --- | --- | --- | --- | --- | --- | --- |
| **Recipient Factors** | | | | | | | | |
|  | **SRR_SCS (n=937)** | **SRR_NMP (Overall) (n=2637)** | **P-value¹** | **On-site**  **(n=2053)** | **P-value²** | **Back-to-Base (n=584)** | **P-value³** | **P-value⁴** |
| **Age** | 57.13(9.98) | 56.75(10.85) | 0.33 | 56.69(10.92) | 0.28 | 56.96(10.62) | 0.75 | 0.60 |
| **Height (cm)** | 172.68(10.47) | 171.78(10.33) | **0.02** | 171.7(10.34) | **0.02** | 172.07(10.29) | 0.27 | 0.44 |
| **Weight (kg)** | 86.95(19.74) | 86.66(20.2) | 0.71 | 86.36(20.06) | 0.45 | 87.74(20.66) | 0.46 | 0.15 |
| **BMI (kg/m^2^)** | 29.1(5.88) | 29.27(5.93) | 0.44 | 29.21(5.94) | 0.64 | 29.5(5.9) | 0.19 | 0.29 |
| **Calculated MELD Score** | 18.73(6.74) | 19.12(7.51) | 0.14 | 19.26(7.7) | 0.05 | 18.62(6.79) | 0.76 | 0.05 |
| **Preservation Time (hours)** | 5.61(1.62) | 16.82(5.68) | **<0.01** | 16.53(5.83) | **<0.01** | 17.83(4.99) | **<0.01** | **<0.01** |
| **Gender (%)** |  |  | 0.38 |  | 0.12 |  | 0.85 | 0.26 |
| *Male* | 614 (65.5) | 1664 (63.1) |  | 1284 (62.5) |  | 380 (65.1) |  |  |
| *Female* | 323 (34.5) | 973 (36.9) |  | 769 (37.5) |  | 204 (34.9) |  |  |
| **Ethnicity (%)** |  |  | **<0.01** |  | **<0.01** |  | 0.91 | **<0.01** |
| *Caucasian* | 722 (77.1) | 1885 (71.5) |  | 1438 (70.0) |  | 447 (76.5) |  |  |
| *African American* | 39 (4.2) | 100 (3.8) |  | 75 (3.7) |  | 25 (4.3) |  |  |
| *Hispanic* | 138 (14.7) | 467 (17.7) |  | 374 (18.2) |  | 93 (15.9) |  |  |
| *Asian* | 26 (2.8) | 86 (3.3) |  | 73 (3.6) |  | 13 (2.2) |  |  |
| *Native American/Other* | 12 (1.3) | 99 (3.8) |  | 93 (4.5) |  | 6 (1.0) |  |  |
| **ABO (%)** |  |  | 0.58 |  | 0.77 |  | 0.60 | 0.13 |
| *A* | 367 (39.2) | 1041 (39.5) |  | 829 (40.4) |  | 212 (36.3) |  |  |
| *AB* | 26 (2.8) | 73 (2.8) |  | 59 (2.9) |  | 14 (2.4) |  |  |
| *B* | 78 (8.3) | 232 (8.8) |  | 185 (9.0) |  | 47 (8.0) |  |  |
| *O* | 466 (49.7) | 1291 (49.0) |  | 980 (47.7) |  | 311 (53.3) |  |  |
| **Diabetes (%)** |  |  | **<0.01** |  | **0.01** |  | **<0.01** | 0.14 |
| *Yes* | 265 (28.3) | 890 (33.8) |  | 678 (33.0) |  | 212 (36.3) |  |  |
| *No* | 672 (71.7) | 1747 (66.2) |  | 1375 (67.0) |  | 372 (63.7) |  |  |
| **Portal Vein Thrombosis (%)** |  |  | 0.09 |  | 0.06 |  | 0.22 | 0.38 |
| *Yes* | 123 (13.1) | 397 (15.1) |  | 307 (15.0) |  | 90 (15.4) |  |  |
| *No* | 814 (86.9) | 2232 (84.6) |  | 1738 (84.7) |  | 494 (84.6) |  |  |
| *Unknown* | 0 (0.0) | 8 (0.3) |  | 8 (0.4) |  | 0 (0.0) |  |  |
| **Ascites at Transplant (%)** |  |  | 0.58 |  | 0.68 |  | 0.62 | 0.48 |
| *None* | 214 (22.8) | 609 (23.1) |  | 485 (23.6) |  | 124 (21.2) |  |  |
| *Mild* | 431 (46.0) | 1243 (47.1) |  | 960 (46.8) |  | 283 (48.5) |  |  |
| *Moderate* | 292 (31.2) | 785 (29.8) |  | 608 (29.6) |  | 177 (30.3) |  |  |
| **Encephalopathy at Transplant (%)** |  |  | 0.90 |  | 0.39 |  | 0.12 | **0.02** |
| *Grade 1-2* | 848 (90.5) | 2379 (90.2) |  | 1837 (89.5) |  | 542 (92.8) |  |  |
| *Grade 3-4* | 89 (9.5) | 258 (9.8) |  | 216 (10.5) |  | 42 (7.2) |  |  |
| **Previous Abdominal Surgery (%)** |  |  | 0.06 |  | 0.08 |  | 0.30 | **0.00** |
| *Yes* | 494 (52.7) | 1425 (54.0) |  | 1128 (54.9) |  | 297 (50.9) |  |  |
| *No* | 432 (46.1) | 1166 (44.2) |  | 882 (43.0) |  | 284 (48.6) |  |  |
| *Unknown* | 11 (1.2) | 46 (1.7) |  | 43 (2.1) |  | 3 (0.5) |  |  |
| **On Dialysis at Transplant (%)** |  |  | 0.08 |  | **0.04** |  | 0.96 | **0.05** |
| *Yes* | 15 (1.6) | 72 (2.7) |  | 64 (3.1) |  | 8 (1.4) |  |  |
| *No* | 916 (97.8) | 2553 (96.8) |  | 1980 (96.4) |  | 573 (98.1) |  |  |
| *Unknown* | 6 (0.6) | 12 (0.5) |  | 9 (0.4) |  | 3 (0.5) |  |  |
| **On Ventilator at Transplant (%)** |  |  | 0.54 |  | 0.17 |  | 0.53 | 0.08 |
| *Yes* | 2 (0.2) | 12 (0.5) |  | 12 (0.6) |  | 0 (0.0) |  |  |
| *No* | 935 (99.8) | 2625 (99.5) |  | 2041 (99.4) |  | 584 (100.0) |  |  |
| **History of Spontaneous Bacterial Peritonitis** |  |  | 0.84 |  | 0.70 |  | 1.00 | 0.78 |
| *Yes* | 88 (9.4) | 260 (9.9) |  | 206 (10.0) |  | 54 (9.2) |  |  |
| *No* | 849 (90.6) | 2375 (90.1) |  | 1845 (89.9) |  | 530 (90.8) |  |  |
| *Unknown* | 0 (0.0) | 2 (0.1) |  | 2 (0.1) |  | 0 (0.0) |  |  |
| **Medical Condition at Transplant (%)** |  |  | **0.04** |  | **0.01** |  | 0.41 | **<0.01** |
| *Home* | 804 (85.8) | 2205 (83.6) |  | 1690 (82.3) |  | 515 (88.2) |  |  |
| *Hospitalized* | 115 (12.3) | 341 (12.9) |  | 281 (13.7) |  | 60 (10.3) |  |  |
| *ICU* | 18 (1.9) | 91 (3.5) |  | 82 (4.0) |  | 9 (1.5) |  |  |
| **MELD Exception Status (%)** |  |  | **<0.01** |  | **<0.01** |  | **<0.01** | 0.21 |
| *No exception* | 677 (72.3) | 2569 (97.4) |  | 2006 (97.7) |  | 563 (96.4) |  |  |
| *HCC Exception* | 37 (3.9) | 17 (0.6) |  | 12 (0.6) |  | 5 (0.9) |  |  |
| *Non-HCC Exception* | 223 (23.8) | 51 (1.9) |  | 35 (1.7) |  | 16 (2.7) |  |  |
| **Hepatitis C NAT (%)** |  |  | 0.21 |  | 0.16 |  | 0.56 | 0.73 |
| *Positive* | 34 (3.6) | 69 (2.6) |  | 52 (2.5) |  | 17 (2.9) |  |  |
| *Negative* | 903 (96.4) | 2567 (97.3) |  | 2000 (97.4) |  | 567 (97.1) |  |  |
| *Unknown* | 0 (0.0) | 1 (0.0) |  | 1 (0.0) |  | 0 (0.0) |  |  |
| **Working for Income (%)** |  |  | **<0.01** |  | **0.01** |  | **0.01** | 0.53 |
| *Yes* | 271 (28.9) | 879 (33.3) |  | 682 (33.2) |  | 197 (33.7) |  |  |
| *No* | 649 (69.3) | 1739 (65.9) |  | 1354 (66.0) |  | 385 (65.9) |  |  |
| *Unknown* | 17 (1.8) | 19 (0.7) |  | 17 (0.8) |  | 2 (0.3) |  |  |
| **TIPS Shunt (%)** |  |  | **<0.01** |  | **<0.01** |  | 0.83 | **<0.01** |
| *Yes* | 124 (13.2) | 313 (11.9) |  | 232 (11.3) |  | 81 (13.9) |  |  |
| *No* | 797 (85.1) | 2200 (83.4) |  | 1705 (83.0) |  | 495 (84.8) |  |  |
| *Unknown* | 16 (1.7) | 124 (4.7) |  | 116 (5.7) |  | 8 (1.4) |  |  |
| **Diagnosis at Transplant (%)** |  |  | **0.01** |  | **<0.01** |  | 0.57 | **<0.01*** |
| *Acute Liver Failure* | 2 (0.2) | 14 (0.5) |  | 14 (0.7) |  | 0 (0.0) |  |  |
| *Alcohol Cirrhosis* | 317 (33.8) | 792 (30.0) |  | 604 (29.4) |  | 188 (32.2) |  |  |
| *Alcohol Hepatitis* | 21 (2.2) | 88 (3.3) |  | 69 (3.4) |  | 19 (3.3) |  |  |
| *Cholestatic Disease* | 47 (5.0) | 191 (7.2) |  | 166 (8.1) |  | 25 (4.3) |  |  |
| *HCV* | 51 (5.4) | 98 (3.7) |  | 72 (3.5) |  | 26 (4.5) |  |  |
| *MASH* | 201 (21.5) | 560 (21.2) |  | 414 (20.2) |  | 146 (25.0) |  |  |
| *Malignancy* | 188 (20.1) | 548 (20.8) |  | 436 (21.2) |  | 112 (19.2) |  |  |
| *Other* | 110 (11.7) | 346 (13.1) |  | 278 (13.5) |  | 68 (11.6) |  |  |
| **Donor Factors** | | | | | | | | |
|  | **SRR_SCS (n=937)** | **SRR_NMP (Overall) (n=2637)** | **P-value¹** | **On-site (n=2053)** | **P-value²** | **Back-to-Base (n=584)** | **P-value³** | **P-value⁴** |
| **Age** | 39.96(13.44) | 47.15(13.95) | **<0.01** | 47.52(13.88) | **<0.01** | 45.83(14.13) | **<0.01** | **0.01** |
| **Height** | 171.53(10.52) | 170.66(11.1) | **0.03** | 170.47(11.16) | **0.01** | 171.34(10.87) | 0.74 | 0.09 |
| **Weight** | 80.3(19.21) | 84.76(21.98) | **<0.01** | 85.38(22.26) | **<0.01** | 82.56(20.83) | **0.03** | **<0.01** |
| **BMI** | 27.28(6.17) | 29.1(7.05) | **<0.01** | 29.38(7.17) | **<0.01** | 28.1(6.53) | **0.01** | **<0.01** |
| **AST** | 74.44(102.54) | 77.44(141.38) | 0.49 | 71.94(100.81) | 0.53 | 96.8(232.65) | **0.03** | **0.01** |
| **ALT** | 76.14(128.84) | 73.51(164.47) | 0.62 | 70.06(153.67) | 0.26 | 85.65(197.48) | 0.30 | 0.08 |
| **Total Bilirubin** | 0.68(0.55) | 0.64(0.54) | 0.10 | 0.64(0.53) | 0.06 | 0.67(0.57) | 0.65 | 0.31 |
| **Liver DSRI** | 34.96(36.53) | 51.4(71.69) | **<0.01** | 49.35(55.12) | **<0.01** | 58.57(111.47) | **<0.01** | 0.05 |
| **Distance** | 129.17(178.14) | 213.75(289.02) | **<0.01** | 224.72(304.5) | **<0.01** | 175.2(222.34) | **<0.01** | **<0.01** |
| **Gender (%)** |  |  | 0.37 |  | 0.25 |  | 0.78 | 0.52 |
| *Male* | 610 (65.1) | 1668 (63.3) |  | 1292 (62.9) |  | 376 (64.4) |  |  |
| *Female* | 327 (34.9) | 969 (36.7) |  | 761 (37.1) |  | 208 (35.6) |  |  |
| **Ethnicity (%)** |  |  | **<0.01** |  | **<0.01** |  | 0.10 | 0.11 |
| *Caucasian* | 680 (72.6) | 1943 (73.7) |  | 1492 (72.7) |  | 451 (77.2) |  |  |
| *African American* | 148 (15.8) | 304 (11.5) |  | 240 (11.7) |  | 64 (11.0) |  |  |
| *Hispanic* | 75 (8.0) | 277 (10.5) |  | 226 (11.0) |  | 51 (8.7) |  |  |
| *Asian* | 25 (2.7) | 59 (2.2) |  | 47 (2.3) |  | 12 (2.1) |  |  |
| *Native American/Other* | 9 (1.0) | 54 (2.0) |  | 48 (2.3) |  | 6 (1.0) |  |  |
| **ABO (%)** |  |  | 0.52 |  | 0.43 |  | 0.21 | 0.17 |
| *A* | 379 (40.4) | 1028 (39.0) |  | 820 (39.9) |  | 208 (35.6) |  |  |
| *AB* | 9 (1.0) | 44 (1.7) |  | 36 (1.8) |  | 8 (1.4) |  |  |
| *B* | 82 (8.8) | 226 (8.6) |  | 178 (8.7) |  | 48 (8.2) |  |  |
| *O* | 467 (49.8) | 1339 (50.8) |  | 1019 (49.6) |  | 320 (54.8) |  |  |
| **Diabetes (%)** |  |  | **<0.01** |  | **<0.01** |  | **<0.01** | 0.91 |
| *Yes* | 98 (10.5) | 467 (17.7) |  | 367 (17.9) |  | 100 (17.1) |  |  |
| *No* | 828 (88.4) | 2148 (81.5) |  | 1669 (81.3) |  | 479 (82.0) |  |  |
| *Unknown* | 11 (1.2) | 22 (0.8) |  | 17 (0.8) |  | 5 (0.9) |  |  |
| **Cause of Death (%)** |  |  | 0.32 |  | 0.12 |  | 0.99 | 0.33 |
| *Anoxia* | 506 (54.0) | 1382 (52.4) |  | 1072 (52.2) |  | 310 (53.1) |  |  |
| *CVA* | 199 (21.2) | 622 (23.6) |  | 495 (24.1) |  | 127 (21.7) |  |  |
| *Trauma* | 190 (20.3) | 491 (18.6) |  | 371 (18.1) |  | 120 (20.5) |  |  |
| *Other* | 42 (4.5) | 142 (5.4) |  | 115 (5.6) |  | 27 (4.6) |  |  |
| **IV Drug Use (%)** |  |  | 0.28 |  | 0.13 |  | 0.71 | 0.31 |
| *Yes* | 108 (11.5) | 256 (9.7) |  | 190 (9.3) |  | 66 (11.3) |  |  |
| *No* | 815 (87.0) | 2331 (88.4) |  | 1825 (88.9) |  | 506 (86.6) |  |  |
| *Unknown* | 14 (1.5) | 50 (1.9) |  | 38 (1.9) |  | 12 (2.1) |  |  |
| **HIV NAT (%)** |  |  | 0.16 |  | 0.38 |  | 0.62 | 0.53 |
| *Positive* | 1 (0.1) | 0 (0.0) |  | 0 (0.0) |  | 0 (0.0) |  |  |
| *Negative* | 936 (99.9) | 2634 (99.9) |  | 2051 (99.9) |  | 583 (99.8) |  |  |
| *Unknown* | 0 (0.0) | 3 (0.1) |  | 2 (0.1) |  | 1 (0.2) |  |  |
| **Hepatitis C NAT (%)** |  |  | 0.21 |  | 0.16 |  | 0.56 | 0.73 |
| *Positive* | 34 (3.6) | 69 (2.6) |  | 52 (2.5) |  | 17 (2.9) |  |  |
| *Negative* | 903 (96.4) | 2567 (97.3) |  | 2000 (97.4) |  | 567 (97.1) |  |  |
| *Unknown* | 0 (0.0) | 1 (0.0) |  | 1 (0.0) |  | 0 (0.0) |  |  |
| **Heavy Alcohol Use (%)** |  |  | **0.02** |  | 0.20 |  | **0.05** | 0.21 |
| *Yes* | 174 (18.6) | 566 (21.5) |  | 438 (21.3) |  | 128 (21.9) |  |  |
| *No* | 734 (78.3) | 1975 (74.9) |  | 1547 (75.4) |  | 428 (73.3) |  |  |
| *Unknown* | 29 (3.1) | 96 (3.6) |  | 68 (3.3) |  | 28 (4.8) |  |  |
| **History of Hypertension (%)** |  |  | **<0.01** |  | **<0.01** |  | **<0.01** | 0.12 |
| *Positive* | 293 (31.3) | 1179 (44.7) |  | 935 (45.5) |  | 244 (41.8) |  |  |
| *Negative* | 633 (67.6) | 1433 (54.3) |  | 1096 (53.4) |  | 337 (57.7) |  |  |
| *Unknown* | 11 (1.2) | 25 (0.9) |  | 22 (1.1) |  | 3 (0.5) |  |  |
| **MACROSTEATOSIS (%)** |  |  | **<0.01** |  | **<0.01** |  | **<0.01** | **<0.01** |
| *No Biopsy* | 717 (76.5) | 1336 (50.7) |  | 987 (48.1) |  | 349 (59.8) |  |  |
| *1 (<30)* | 209 (22.3) | 1232 (46.7) |  | 1014 (49.4) |  | 218 (37.3) |  |  |
| *2(>=30)* | 11 (1.2) | 69 (2.6) |  | 52 (2.5) |  | 17 (2.9) |  |  |

**Abbreviations:** SRR_SCS, super-rapid recovery with static cold storage; SRR_NMP, super-rapid recovery with normothermic machine perfusion; On-site, SRR-NMP performed on-site; Back-to-Base, SRR-NMP performed at the transplant center (“back-to-base”).

¹ P value compares SRR_SCS vs SRR_NMP (Overall)
² P value compares SRR_SCS vs On-site
³ P value compares SRR_SCS vs Back-to-Base
⁴ P value compares On-site vs Back-to-Base

Continuous variables are presented as mean ± SD and compared using Student’s t-test.
Categorical variables are presented as n (%) and compared using chi-square or Fisher’s exact test, where appropriate.
* P value using Monte Carlo test

| **Supplemental Table 2: Baseline recipient and donor characteristics by NRP-SCS and NRP-NMP in DCD liver transplantation, United States, 2022–2024** | | | |
| --- | --- | --- | --- |
| **Recipient Factors** | | | |
|  | **NRP_SCS (n=560)** | **NRP_NMP (n=498)** | **P-value** |
| **Age** | 56.28(10.39) | 57.99(11.04) | 0.01 |
| **Height (cm)** | 172.59(10.57) | 172.41(9.81) | 0.77 |
| **Weight (kg)** | 88.06(20.42) | 87.61(20.11) | 0.72 |
| **BMI (kg/m^2^)** | 29.46(5.79) | 29.39(5.94) | 0.84 |
| **Calculated MELD Score** | 19.33(6.82) | 18.67(7.47) | 0.14 |
| **Preservation Time (hours)** | 5.13(1.57) | 15.18(6.65) | <0.01 |
| **Gender (%)** |  |  | 0.43 |
| *Male* | 371 (66.3) | 320 (64.3) |  |
| *Female* | 189 (33.8) | 178 (35.7) |  |
| **Ethnicity (%)** |  |  | <0.01 |
| *Caucasian* | 420 (75.0) | 389 (78.1) |  |
| *African American* | 21 (3.8) | 17 (3.4) |  |
| *Hispanic* | 84 (15.0) | 70 (14.1) |  |
| *Asian* | 10 (1.8) | 12 (2.4) |  |
| *Native American/Other* | 25 (4.5) | 10 (2.0) |  |
| **ABO (%)** |  |  | 0.53 |
| *A* | 232 (41.4) | 183 (36.7) |  |
| *AB* | 7 (1.3) | 16 (3.2) |  |
| *B* | 48 (8.6) | 49 (9.8) |  |
| *O* | 273 (48.8) | 250 (50.2) |  |
| **Diabetes (%)** |  |  | <0.01 |
| *Yes* | 155 (27.7) | 167 (33.5) |  |
| *No* | 405 (72.3) | 331 (66.5) |  |
| **Portal Vein Thrombosis (%)** |  |  |  |
| *Yes* | 84 (15.0) | 71 (14.3) |  |
| *No* | 474 (84.6) | 424 (85.1) |  |
| *Unknown* | 2 (0.4) | 3 (0.6) |  |
| **Ascites at Transplant (%)** |  |  | 0.61 |
| *None* | 109 (19.5) | 113 (22.7) |  |
| *Mild* | 278 (49.6) | 228 (45.8) |  |
| *Moderate* | 173 (30.9) | 157 (31.5) |  |
| **Encephalopathy at Transplant (%)** |  |  | 0.84 |
| *Grade 1-2* | 507 (90.5) | 445 (89.4) |  |
| *Grade 3-4* | 53 (9.5) | 53 (10.6) |  |
| **Previous Abdominal Surgery (%)** |  |  | 0.03 |
| *Yes* | 275 (49.1) | 291 (58.4) |  |
| *No* | 277 (49.5) | 198 (39.8) |  |
| *Unknown* | 8 (1.4) | 9 (1.8) |  |
| **On Dialysis at Transplant (%)** |  |  | 0.13 |
| *Yes* | 7 (1.3) | 12 (2.4) |  |
| *No* | 553 (98.8) | 485 (97.4) |  |
| *Unknown* | 0 (0.0) | 1 (0.2) |  |
| **On Ventilator at Transplant (%)** |  |  | 0.25 |
| *Yes* | 3 (0.5) | 0 (0.0) |  |
| *No* | 557 (99.5) | 498 (100.0) |  |
| **History of Spontaneous Bacterial Peritonitis (%)** |  |  | 0.56* |
| *Yes* | 55 (9.8) | 43 (8.6) |  |
| *No* | 504 (90.0) | 455 (91.4) |  |
| *Unknown* | 1 (0.2) | 0 (0.0) |  |
| **Medical Condition at Transplant (%)** |  |  | 0.12* |
| *Home* | 487 (87.0) | 411 (82.5) |  |
| *Hospitalized* | 59 (10.5) | 71 (14.3) |  |
| *ICU* | 13 (2.3) | 16 (3.2) |  |
| *Unknown* | 1 (0.2) | 0 (0.0) |  |
| **MELD Exception Status (%)** |  |  | <0.01 |
| *No exception* | 522 (93.2) | 494 (99.2) |  |
| *HCC Exception* | 7 (1.3) | 2 (0.4) |  |
| *Non-HCC Exception* | 31 (5.5) | 2 (0.4) |  |
| **Hepatitis C NAT (%)** |  |  | 0.50 |
| *Positive* | 21 (3.8) | 13 (2.6) |  |
| *Negative* | 539 (96.3) | 485 (97.4) |  |
| **Working for Income (%)** |  |  | 0.01 |
| *Yes* | 199 (35.5) | 159 (31.9) |  |
| *No* | 360 (64.3) | 329 (66.1) |  |
| *Unknown* | 1 (0.2) | 10 (2.0) |  |
| **TIPS Shunt (%)** |  |  | <0.01 |
| *Yes* | 55 (9.8) | 73 (14.7) |  |
| *No* | 491 (87.7) | 401 (80.5) |  |
| *Unknown* | 14 (2.5) | 24 (4.8) |  |
| **Diagnosis at Transplant (%)** |  |  | 0.21* |
| *Acute Liver Failure* | 1 (0.2) | 1 (0.2) |  |
| *Alcohol Cirrhosis* | 188 (33.6) | 139 (27.9) |  |
| *Alcohol Hepatitis* | 15 (2.7) | 17 (3.4) |  |
| *Cholestatic Disease* | 36 (6.4) | 31 (6.2) |  |
| *HCV* | 17 (3.0) | 19 (3.8) |  |
| *MASH* | 121 (21.6) | 128 (25.7) |  |
| *Malignancy* | 111 (19.8) | 115 (23.1) |  |
| *Other* | 71 (12.7) | 48 (9.6) |  |
| **Donor Factors** | | | |
|  | **NRP_SCS (n=560)** | **NRP_NMP (n=498)** | **P-value** |
| **Age** | 46.81(14.27) | 47.97(15.12) | 0.20 |
| **Height** | 172.51(10.33) | 171.38(10.72) | 0.08 |
| **Weight** | 85.7(21.67) | 84.86(23.05) | 0.54 |
| **BMI** | 28.87(7.27) | 28.89(7.7) | 0.97 |
| **AST** | 79.01(124.07) | 93.69(194.05) | 0.15 |
| **ALT** | 68.66(104.11) | 91.44(253.56) | 0.06 |
| **Total Bilirubin** | 0.78(2.41) | 0.67(0.55) | 0.28 |
| **Liver DSRI** | 53.33(75.43) | 56.25(102.23) | 0.60 |
| **Distance** | 103.02(153.53) | 163.02(244.25) | <0.01 |
| **Gender (%)** |  |  | 0.18 |
| *Male* | 370 (66.1) | 330 (66.3) |  |
| *Female* | 190 (33.9) | 168 (33.7) |  |
| **Ethnicity (%)** |  |  | 0.18 |
| *Caucasian* | 428 (76.4) | 382 (76.7) |  |
| *African American* | 65 (11.6) | 42 (8.4) |  |
| *Hispanic* | 50 (8.9) | 62 (12.4) |  |
| *Asian* | 7 (1.3) | 4 (0.8) |  |
| *Native American/Other* | 10 (1.8) | 8 (1.6) |  |
| **ABO (%)** |  |  |  |
| *A* | 228 (40.7) | 182 (36.5) | 0.18 |
| *AB* | 4 (0.7) | 9 (1.8) |  |
| *B* | 44 (7.9) | 49 (9.8) |  |
| *O* | 284 (50.7) | 258 (51.8) |  |
| **Diabetes (%)** |  |  | 0.36 |
| *Yes* | 98 (17.5) | 89 (17.9) |  |
| *No* | 459 (82.0) | 402 (80.7) |  |
| *Unknown* | 3 (0.5) | 7 (1.4) |  |
| **Cause of Death (%)** |  |  | 0.08 |
| *Anoxia* | 295 (52.7) | 256 (51.4) |  |
| *CVA* | 111 (19.8) | 118 (23.7) |  |
| *Trauma* | 125 (22.3) | 93 (18.7) |  |
| *Other* | 29 (5.2) | 31 (6.2) |  |
| **IV Drug Use (%)** |  |  | 0.31 |
| *Yes* | 65 (11.6) | 40 (8.0) |  |
| *No* | 483 (86.3) | 447 (89.8) |  |
| *Unknown* | 12 (2.1) | 11 (2.2) |  |
| **HIV NAT (%)** |  |  | 0.46 |
| *Negative* | 560 (100.0) | 498 (100.0) |  |
| **Hepatitis C NAT (%)** |  |  | 0.50 |
| *Positive* | 21 (3.8) | 13 (2.6) |  |
| *Negative* | 539 (96.3) | 485 (97.4) |  |
| **Heavy Alcohol Use (%)** |  |  | 0.02 |
| *Yes* | 145 (25.9) | 114 (22.9) |  |
| *No* | 396 (70.7) | 360 (72.3) |  |
| *Unknown* | 19 (3.4) | 24 (4.8) |  |
| **History of Hypertension (%)** |  |  | <0.01 |
| *Positive* | 247 (44.1) | 222 (44.6) |  |
| *Negative* | 306 (54.6) | 268 (53.8) |  |
| *Unknown* | 7 (1.3) | 8 (1.6) |  |
| **MACROSTEATOSIS (%)** |  |  | <0.01 |
| *No Biopsy* | 293 (52.3) | 213 (42.8) |  |
| *1 (<30)* | 255 (45.5) | 262 (52.6) |  |
| *2(>=30)* | 12 (2.1) | 23 (4.6) |  |

**Abbreviations:** DCD, donation after circulatory death; NRP_SCS, normothermic regional perfusion with static cold storage; NRP_NMP, normothermic regional perfusion with normothermic machine perfusion.

Continuous variables are presented as mean ± SD and compared using Student’s t-test.
Categorical variables are presented as n (%) and compared using chi-square or Fisher’s exact test, where appropriate.
* P value compares NRP-NMP vs NRP-SCS using Monte Carlo test.
